# Broad-spectrum host-targeting antivirals for rhinovirus infections: mechanisms and future perspectives

**DOI:** 10.3389/fimmu.2026.1892269

**Published:** 2026-08-18

**Authors:** Wanru Shang, Hongbing Luo, Siqi Li, Shichao Yu, Xinyu Wang, Dongye Fu, Lijun Wang, Fanzheng Meng, Man Gao

**Affiliations:** 1 Department of Pediatric Respiration, Children’s Medical Center, The First Hospital of Jilin University, Changchun, China; 2 Center for Pathogen Biology and Infectious Diseases, The First Hospital of Jilin University, Changchun, China

**Keywords:** host factors, host-pathogen interaction, host-targeting antivirals, immunomodulation, rhinovirus

## Abstract

Rhinovirus is the main pathogen causing common colds, acute exacerbations of asthma, chronic obstructive pulmonary disease and other respiratory diseases. Due to its numerous serotypes and the lack of common conserved viral targets, the development of traditional direct-acting antiviral drugs (DAAs) faces challenges. Host-targeting antivirals (HTAs), as an emerging strategy, provide a new approach to overcome this difficulty by interfering with the host cell factors or pathways necessary for viral replication. This review systematically presents the latest progress in host-targeted rhinovirus therapy by focusing on numerous host factors involved in rhinovirus invasion, attachment, biosynthesis, assembly and release, and immune regulation. Although the prospects are broad, the clinical transformation of this strategy still faces challenges such as safety, delivery efficiency, and individual differences. In conclusion, HTAs are expected to become a transformative treatment strategy for rhinovirus infection, especially for high-risk patient groups.

## 1 Introduction

**Figure 1:**
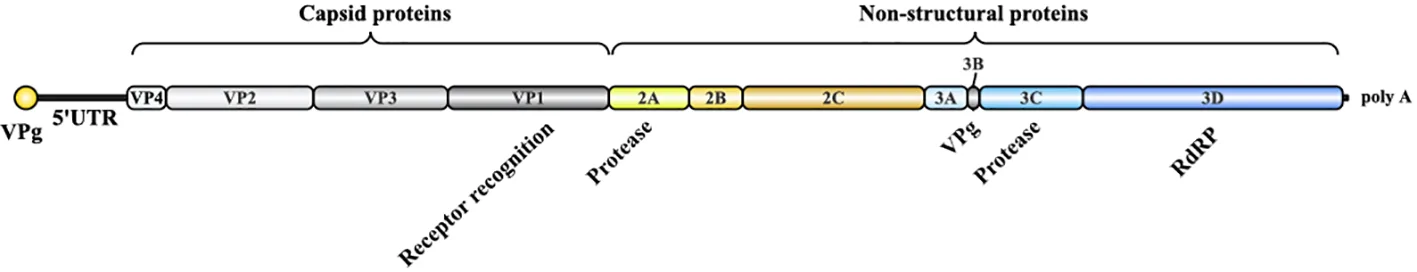
Rhinovirus genomic structure. Rhinovirus belongs to the single-stranded positive-sense RNA viruses, with a total length of approximately 7,200 bp. The capsid protein VP1 is primarily responsible for receptor recognition; VP4, VP2, VP3, and VP1 are processed to form the rhinovirus capsid, while the remaining proteins are processed to generate proteases, VPg and RNA-dependent RNA polymerase (RdRP).

Rhinovirus (RV) is a member of the Enterovirus genus in the Picornaviridae family. It is a non-enveloped virus with approximately 7.2 kb of positive-sense single-stranded RNA (+ssRNA). The viral genome contains only one open reading frame. The 5’-end of the genome contains a non-coding region and is capped by the small viral priming protein (VPg), while the 3’-end is polyadenylated. After the viral proteins are translated, they are cleaved by proteases into 11 proteins, among which the structural proteins VP1, VP2, VP3, and VP4 form the protein capsid, while the non-structural proteins (2A-2C, 3A-3D) participate in viral replication and assembly (1–3). (**Figure 1**). There are currently more than 160 known RV serotypes, which are divided into three main groups: RV-A, RV-B, and RV-C. These distinct serotypes invade host cells via different cell surface receptors: intercellular adhesion molecule-1 (ICAM-1) (most RV-A and all RV-B), members of the low-density lipoprotein receptor (LDLR) family (12 RV-A types), and cadherin-related family member 3 (CDHR3) (RV-C) (4, 5).

As one of the primary pathogens causing the common cold, growing evidence suggests that RV is not only responsible for self-limiting upper respiratory infections, but is also linked to a variety of severe lower respiratory diseases, including wheezing, asthma exacerbations, pneumonia, and chronic obstructive pulmonary disease (COPD) (6, 7). Early-life RV infection is strongly associated with recurrent wheezing and childhood asthma (8, 9). A prospective multicenter study found that newborns infected with RV-C had a considerably higher incidence of recurrent wheezing, which was followed by the development of asthma (10).

At present, the clinical treatment of RV infection mainly focuses on symptomatic and supportive care, such as reducing mucosal edema, inhibiting histamine release and using interferons to regulate the immune system. Although these approaches can alleviate symptoms, they cannot inhibit RV replication or significantly shorten the infection course. To date, there are no approved human rhinovirus (HRV) vaccines or antiviral drugs. Research and development efforts have mainly focused on direct-acting antivirals (DAAs), including small-molecule capsid inhibitors such as Pleconaril, Pyrazolopyrimidines, and Pirodavir, as well as protease inhibitors targeting the 3C protease, such as Rupintrivir, SG85, and AG7404 (11–16).

The viral polymerase is the core target for the development of DAAs. However, this strategy has obvious limitations. Firstly, the structures of polymerases from different viruses and genotypes vary significantly, making it difficult for drugs targeting specific polymerases to be widely applicable (17). Secondly, because DAAs directly target viral proteins, their selective pressure will drive adaptive genetic mutations in the virus during replication, ultimately leading to significant drug resistance to DAAs (18). Like many other respiratory viruses, a notable feature of RV is its rapid replication and high mutation rate, resulting in significant genetic diversity. Therefore, these DAAs are prone to developing resistance due to viral mutations and have varying effects across different RV types (19).

In response to these limitations, Host-targeting antivirals (HTAs) have emerged as a promising alternative strategy. Unlike conventional approaches that target viral components directly, HTAs exert broad-spectrum antiviral activity by modulating key host cellular factors and pathways essential for viral replication, thereby raising the genetic barrier to resistance (20). For example, Ribavirin, a clinically approved antiviral agent, is commonly recognized for its ability to modulate host cellular pathways to achieve antiviral activity. Its primary mechanism involves inhibiting inosine monophosphate dehydrogenase (IMPDH), thereby disrupting the synthesis of viral nucleic acids (21, 22). Currently, it is used to manage infections caused by respiratory syncytial virus (RSV) and hepatitis C virus (HCV), among other viral diseases (23, 24), particularly in patients with hematologic malignancies and those who have undergone hematopoietic stem cell transplantation (25, 26). However, intervention strategies targeting host factors may be toxic. The reason is that such targets usually play an important role in the basic physiological activities of cells. How to balance the relationship between normal host physiological functions and antiviral effects is also a key issue that needs to be urgently addressed. This review summarizes recent advances in HTAs for RV infections and aims to provide a reference for the development of novel treatment strategies against this important pathogen (**Table 1**).

**Table 1:** Summary of host-targeting antiviral agents against rhinovirus described in this article.

Host target	Antiviral agents	Model system	Antiviral outcome	Mode of action	Major safety limitations	Translation status
ICAM-1	RRMA	Human clinical trial	Poor antiviral effect	Target cellular ICAM-1 (29)	Narrow therapeutic window	Development Stalled
	Tremacamra	Human clinical trial; *In vitro* cell systems	Reduce the infection titer and clinical symptoms	Decoy receptors (30, 31)	Narrow therapeutic window	Development Stalled
	14C11	Murine model	Effective against specific strains (HRV-14, HRV-16)	Target cellular ICAM-1 (33)	Unproven broad-spectrum efficacy (limited to few serotypes); Human respiratory safety unknown	Preclinical (for rhinovirus)
	Cilomilast	*In vitro* cell systems; Human clinical trial (COPD patients)	Reduce the release of inflammatory factors caused by rhinovirus infection	Down-regulate the expression level of ICAM-1 (34)	A range of gastrointestinal symptoms such as nausea, vomiting and dyspepsia (36, 37); Unfavorable safety profile for general population prophylaxis	Preclinical (for rhinovirus)
	EPs 7630 (Umckaloabo®)	Human clinical trial; *In vitro* cell systems	Reduce clinical symptoms and viral infection	Down-regulate the expression of ICOS, ICOSL, C1qR and ICAM-1 (41, 42)	Favorable safety profile	Approved (Europe, Asia, etc. for aRTIs); Preclinical (for rhinovirus)
LDLR	Recombinant soluble fragments of LDLR	*In vitro* cell systems	Reduce the infection titer	Decoy receptors (46)	A high risk of inducing severe hypercholesterolemia and accelerating atherosclerosis (49, 50)	Preclinical (for rhinovirus)
	4H30	*In vitro* cell systems	Block viral entry and replication(Effective against LDLR-using strains)	Target the LDLR (57)	Lipid metabolism disturbances	Preclinical (for rhinovirus)
CDHR3	No reports yet	No reports yet	No reports yet	No reports yet	No reports yet	No reports yet
Sialic acid receptors	HEX17 (Neumifil)	*In vitro* cell systems	Reduce viral infection	Interact with ICAM-1and LDLR (65)	No reports yet	Preclinical (for rhinovirus)
Acidic organelles	Niclosamide	*In vitro* cell systems	Inhibit viral endosomal acidification to block uncoating and RNA release	Neutralize the pH of acidic organelles (69)	Potent cytotoxic/proliferative inhibitory effects (71); Induce non-canonical autophagy by triggering non-canonical LC3 lipidation (72)	Preclinical (for rhinovirus)
	P9R	*In vitro* cell systems	Inhibit viral endosomal acidification to block uncoating and RNA release	Inhibit viral endosomal acidification (74)	May have hemolytic activity (75)	Preclinical (for rhinovirus)
Oxysterol-binding protein (OSBP)	Itraconazole	Murine model	Reduce viral loads and release of pro-inflammatory mediators	Disrupt lipid transfer function and suppressing viral genome replication (82)	Prolong the QT interval and triggering malignant arrhythmias (83–85)	Preclinical (for rhinovirus)
Endogenous cholesterol metabolites	25OHC and 27OHC	*In vitro* cell systems	Reduce viral titers and protects against virus-induced ciliary damage	Induce OSBP delocalization from Golgi vesicles, thereby reducing PI4P on the endoplasmic reticulum (90, 91)	Disrupt the cholesterol homeostasis of cells (the synthesis of pulmonary surfactant, lipid raft structure and signal transduction) (92, 93)	Preclinical (for rhinovirus)
Fatty acid synthase	TVB-3166	*In vitro* cell systems	Reduce the production of HRV-16 infectious progeny	Inhibit viral protein synthesis (95)	Hypoglycemia, weight loss, and hepatic steatosis (96)	Preclinical (for rhinovirus)
HMG-CoA reductase	Simvastatin	*In vitro* cell systems	Attenuate the inflammatory response during rhinovirus infection	Suppress STAT1 phosphorylation in the JAK/STAT signaling pathway (98, 99)	The adverse reactions associated with the use of statins, such as statin-related muscle symptoms (101)	Preclinical (for rhinovirus)
Nuclear proteins (e.g. SFPQ)	No reports yet	No reports yet	No reports yet	No reports yet	No reports yet	No reports yet
Cellular helicases(e.g. eIF4A)	Silvestrol	*In vitro* cell systems	Reduce the infection titer	Impede the assembly of translation initiation complexes (109)	Unclear safety profile for rhinovirus infection	Preclinical (for rhinovirus)
MAPK pathways	No reports yet	No reports yet	No reports yet	No reports yet	Dermatological toxicities, including rash and acneiform dermatitis (117), as well as gastrointestinal disturbances such as nausea, vomiting, and diarrhea (118)	No reports yet
Phosphoinositide kinases	Enviroxime-like compounds	*In vitro* cell systems	Suppress rhinovirus replication	Inhibit PI4KIIIβ (122)	Dose-dependent cytotoxicity	Preclinical (for rhinovirus)
	LY294002	*In vitro* cell systems	Suppress rhinovirus replication	Block Akt phosphorylation (123)	Metabolic abnormalities, including elevated total cholesterol, LDL cholesterol, and triglycerides (127).	Preclinical (for rhinovirus)
	2-DG	Human clinical trial; *In vitro* cell systems; Murine model	Inhibit rhinovirus replication and reduce clinical symptoms	Inhibit the glycolysis (129)	No reports yet	Development stage
RIG-I inflammasome	YVAD	*In vitro* cell systems	Suppress rhinovirus replication and reduce the release of inflammatory factors	Inhibit excessive activation of the RIG-I inflammasome pathway (134)	No reports yet	Preclinical (for rhinovirus)
OLFML3	No reports yet	No reports yet	No reports yet	No reports yet	No reports yet	No reports yet

## 2 Host factors targeting the viral entry stage

The initial step in the RV infection cycle entails the attachment of viral particles to specific receptors on the host cell surface, followed by endocytosis and subsequent uncoating. HTAs targeting this stage primarily aim to disrupt virus–receptor interactions and the associated intracellular processes.

### 2.1 Inhibition of viral attachment

#### 2.1.1 Main cell surface receptors

Different RV serotypes employ distinct cell surface receptors for host cell entry, rendering these receptors promising natural targets for HTAs. As the receptor for the large majority of RV, intercellular adhesion molecule-1 (ICAM-1) is a transmembrane glycoprotein belonging to the immunoglobulin superfamily, which is widely expressed on epithelial cells, endothelial cells, and various immune cells (27, 28).

Early studies demonstrated that the monoclonal antibody RRMA, which targets cellular ICAM-1, produced suboptimal efficacy in preventing experimental RV infection in humans (29). Subsequently, researchers developed tremacamra (formerly known as BIRR4), a soluble recombinant truncated form of ICAM-1 lacking the transmembrane and intracellular domains. *In vitro* cellular assays, along with a randomized, double-blind, placebo-controlled clinical trial, showed that tremacamra effectively reduced RV infection titers and mitigated the severity of RV-induced common cold symptoms (30, 31). However, despite these positive clinical signals, the further development of ICAM-1 blockers has stalled, likely due to concerns over the narrow therapeutic window.

Critically, ICAM-1 is not merely a viral portal but is indispensable for host immunity that plays critical roles in mediating cell-cell adhesion, facilitating leukocyte recruitment, and functioning as a co-stimulatory molecule by binding to lymphocyte function-associated antigen-1 (LFA-1) on T cells to support immune defense (27, 28, 32). Systemic inhibition of ICAM-1 therefore carries a significant risk of impairing immune surveillance and exacerbating inflammation. This safety concern underscores the need for agents that can distinguish between viral binding and physiological ligand interactions. In this context, a mouse anti-human ICAM-1 antibody (14C11) was identified that topically or systemically blocks entry of specific strains (HRV-14, HRV-16) in mouse models without inhibiting the ICAM-1/LFA-1 interaction (33). While this suggests a theoretically viable strategy to uncouple antiviral activity from immunosuppression, several translational hurdles remain: the efficacy of this antibody against the broad spectrum of RV serotypes is unproven, and its safety profile in human respiratory tissues has yet to be established. Thus, while 14C11 represents a promising proof of concept for ICAM-1 targeting, it remains an early-stage candidate rather than a clinically validated solution.

Cilomilast is an oral selective phosphodiesterase-4 (PDE4) inhibitor. Recent *in vitro* data have shown that Cilomilast significantly inhibits the expression level of ICAM-1 induced by RV16 infection and effectively reduces the release of the chemokine CXCL8 (34). A multicenter clinical study conducted in patients with COPD has also confirmed that oral Cilomilast can not only maintain lung function but also reduce the risk of COPD exacerbations during a 24-week treatment period (35). However, systemic PDE4 inhibition is frequently associated with dose-limiting adverse effects, including a range of gastrointestinal symptoms such as nausea, vomiting and dyspepsia (36, 37). Consequently, while Cilomilast represents a promising adjunctive therapy for preventing exacerbations in patients with underlying airway diseases, its utility as a broad-spectrum antiviral for the general population is limited by its unfavorable safety profile. Future strategies may require the development of inhaled PDE4 inhibitors to maximize local efficacy while minimizing systemic toxicity.

EPs 7630 (also known as Umckaloabo^®^), a patented liquid herbal preparation derived from the roots of the traditional medicinal plant Pelargonium sidoides, has been approved in numerous countries across Europe, Asia, Australia, and South America for the treatment of acute respiratory tract infections (aRTIs) due to its demonstrated clinical efficacy and favorable safety profile (38). Clinical evidence-based studies have confirmed the therapeutic value of EPs 7630: it can significantly shorten the duration of the common cold in adults and alleviate clinical symptoms (39). For special populations, such as children with asthma, continuous use of EPs 7630 for 5 days can significantly reduce the frequency of asthma attacks (40). The mechanism of action of EPs 7630 against RV has been preliminarily clarified through *in vitro* cell experiments. On the one hand, EPs 7630 regulates the function of human bronchial epithelial cells (hBECs) by down-regulating the expression of inducible the co-stimulatory factor (ICOS), its ligand ICOSL and the cell surface calreticulin (C1qR), thereby reducing RV infection (41). On the other hand, EPs 7630 down-regulates ICAM-1, reducing the virus’s binding sites on host cells, and significantly up-regulates the expression of β-defensin-1 (a small antimicrobial peptide mainly produced by epithelial cells), further enhancing the host’s resistance to RV infection (42). Additionally, EPs 7630 exhibits potent antiviral activity against a broad spectrum of common respiratory viruses, including influenza A virus strains (H1N1 and H3N2), RSV, human coronaviruses, parainfluenza viruses, and SARS-CoV-2 (43, 44). Therefore, as a natural herbal preparation targeting host factors, EPs 7630 demonstrates a low propensity for resistance development and a favorable safety profile, indicating broad prospects for clinical application.

Subgroup RV-A employ receptors that belong to the low-density lipoprotein receptor (LDLR) family to attach to the airway epithelial cells, such as LDLR itself, very low-density lipoprotein receptor (VLDLR), and other related members. LDLR is a multi-domain transmembrane protein that plays a critical role in maintaining cellular cholesterol homeostasis by mediating the endocytic uptake of circulating low-density lipoproteins (45). Marlovits TC et al. demonstrated for the first time that recombinant soluble fragments of LDLR can act as decoy receptor to effectively inhibit a range of HRV subtypes *in vitro (*46). This promising antiviral potential has been corroborated by multiple subsequent studies (47, 48). However, translating LDLR-targeted therapies faces formidable hurdles. Firstly, safety concerns are paramount. Given LDLR’s critical role in systemic lipid metabolism, systemic blockade or depletion of LDLR carries a high risk of inducing severe hypercholesterolemia and accelerating atherosclerosis (49, 50). Secondly, there are still pharmacokinetic limitations. Soluble decoy proteins typically exhibit short plasma half-lives and poor bioavailability in the respiratory tract when administered systemically (51, 52), while developing effective inhaled formulations presents significant technical challenges regarding protein stability and mucosal penetration. Consequently, despite promising *in vitro* data, LDLR-targeted strategies remain confined to the early discovery phase, with no current candidates advancing to clinical trials due to this unfavorable risk-benefit profile.

Defensins are a class of cationic, cysteine-rich peptides classified as α-defensins, β-defensins, and θ-defensins based on disulfide bond positioning (53). These defensins play essential roles in various biological processes, including antimicrobial activity, immunomodulation, antitumor effects, and antiviral functions (54). Human β-defensins are widely expressed in multiple organs, particularly in respiratory epithelial cells. Human β-defensin 2 (HBD2) has been shown to exhibit antiviral activity against RV, RSV, and other respiratory pathogens (55, 56). The 4H30 (a synthetic peptide derived from HBD2) specifically targets the LDLR, thereby effectively blocking viral entry and suppressing viral replication in nasal organoids and cardiomyocytes. However, it shows no efficacy against HRV-A16, suggesting that 4H30 is unable to interfere with the human intercellular adhesion molecule-1 (hICAM-1) receptor to exert antiviral effects (57). While LDLR is an antiviral target due to its role in viral entry, its central function in host lipid metabolism cannot be overlooked. Consequently, systemic targeting of this receptor may cause metabolic disturbances, which could limit its clinical applicability.

Cadherin-related family member 3 (CDHR3), the receptor for HRV-C, harbors two alleles (G and A) at the single-nucleotide polymorphism site rs6967330. The A allele has been identified as a susceptibility locus for recurrent and exacerbated asthma in early childhood and is significantly associated with an increased risk of severe asthma exacerbations (58, 59). While *in vitro* cell experiments have demonstrated that the absence of the CDHR3 protein reduces HRV-C15 infection of primary airway epithelial cells (60), therapeutic translation remains highly speculative. Although Keng et al. successfully cleared Enterovirus A71 (EV-A71) RNA using an AAV-delivered CRISPR-Cas13 system in preclinical models (61), the significant genomic divergence between EV-A71 and RV-C precludes direct translation of these gRNA sequences. Moreover, the native physiological function of CDHR3 in human airway homeostasis is not fully elucidated, so permanent ablation or modulation via gene editing could have unforeseen consequences on epithelial integrity. Future efforts should prioritize elucidating the biological mechanisms of CDHR3 and developing short-acting small-molecule inhibitors to assess the feasibility of this target.

#### 2.1.2 Sialic acid receptors

Sialic acid, a nine-carbon monosaccharide featuring a ketone acid functional group, is predominantly located at the terminal positions of glycoproteins and glycosphingolipids. It plays a critical role in mediating cell surface anti-adhesion properties and facilitating intercellular signaling (62). Accumulating evidence indicates that sialic acid acts as an auxiliary receptor for RV-C, promoting viral attachment to host cells through interactions with glycosylation sites on CDHR3 (63, 64). HEX17 (also known as Neumifil) is a first-in-class intranasal carbohydrate-binding module (CBM) that exhibits high affinity for sialic acid. In an *in vitro* cell infection study, HEX17 could reduce RV infection (65) by interacting with ICAM-1 and LDLR. Furthermore, HEX17 has demonstrated effectiveness against other respiratory viruses, including RSV and SARS-CoV-2 (65, 66), suggesting potential broad-spectrum antiviral activity against diverse respiratory pathogens that utilize sialic acid receptors for entry. It should be noted that the long-term safety profile of altering local sialic acid interactions in the airway remains to be fully elucidated. Consequently, while HEX17 represents a promising host-directed strategy, its clinical efficacy and true broad-spectrum potential require validation in human challenge trials.

### 2.2 Interfering with virus endocytosis and uncoating

Following receptor binding, RV enters cells via clathrin-mediated endocytosis. Uncoating is triggered in the acidic environment of the endosome through receptor-mediated interactions, ultimately releasing the RNA into the cytoplasm (67). This process is tightly regulated by many host factors, including endosomes and lysosomes.

Niclosamide, an approved anthelmintic drug, exhibits broad-spectrum antiviral activity by neutralizing the pH of acidic organelles (e.g., endosomes and lysosomes), disrupting the optimal acidic environment required for viral replication (68). This mechanism is particularly crucial for RV, whose RNA release depends on capsid conformational changes triggered by endosomal acidification. Studies indicate niclosamide protects HeLa cells from HRV 1A, 2, 14, and 16 infections, suggesting it disrupts proton gradients by acting as a proton carrier, thereby effectively blocking rhinovirus RNA release and preventing infection establishment (69). While drug repurposing offers the theoretical advantage of leveraging existing safety data to accelerate development (70), significant translational hurdles specific to niclosamide and respiratory infections remain. *In vitro* experiments have shown that niclosamide has potent cytotoxic or proliferative inhibitory effects (71). It has also been discovered that it may induce non-canonical autophagy by triggering non-canonical LC3 lipidation (72), suggesting that it may have high acute toxicity *in vivo* when the systemic exposure is increased. Secondly, the dose of this drug required to inhibit viral uncoating without causing cytotoxicity is currently unknown. Further *in vivo* experiments, clinical trials, and related research on niclosamide are still needed to systematically evaluate its therapeutic efficacy against rhinovirus infection.

As mentioned earlier, defensins directly inactivate viral replication and target multiple host-virus interaction steps to reduce infectivity in both enveloped and non-enveloped viruses (73). Zhao H et al. have engineered a defensin peptide P9R by substituting weakly positively charged amino acids (histidine and lysine) in P9 (derived from mouseβ-defensin-4) with arginine residues at positions 21 (H→R), 23 (K→R), and 28 (K→R), increasing P9R’s net positive charge (from P9 (+4.7) to P9R (+5.6)) and enhancing its ability to inhibit endosomal acidification. P9R efficiently binds RV particles, selectively inhibiting viral endosomal acidification to block uncoating and RNA release (74). Interestingly, studies on influenza virus (H1N1) revealed no resistance to P9R even after 40 consecutive passages (initial 5μg/ml, later 50μg/ml), with no significant increase in half maximal inhibitory concentration (IC50). In contrast, the control drug zanamivir exhibited marked resistance after only 10 passages (74). These findings provide evidence supporting the concept that binding viruses to basic peptides can broadly inhibit pH-dependent viruses. They also suggest that host-targeted P9R may represent a promising antiviral strategy against pH-dependent respiratory viruses. However, P9R and other cationic peptides face significant obstacles in clinical application. It has been reported that some antimicrobial peptides have hemolytic activity (75) and are easily degraded by proteases (76). Therefore, the antiviral efficacy of this drug still needs to be rigorously verified *in vivo*.

## 3 Inhibitors targeting viral biosynthesis and assembly/release

During viral RNA replication, host-derived RNA polymerase cofactors, ribosomes, and translation initiation factors (e.g., eIF4E) are essential for the synthesis of viral proteins. Concomitantly, host signaling pathways such as PI3K/Akt are activated to modulate the microenvironment conducive to viral replication (77). During viral particle assembly, the host’s vesicular transport machinery and cytoskeletal network facilitate the trafficking of viral structural proteins and the formation of the nucleocapsid. Mature virions are ultimately released via host cell apoptosis or exocytosis, thereby initiating a new cycle of infection (78). Thus, host factors involved in viral replication, assembly, and release represent promising targets for host-directed antiviral therapies.

### 3.1 Lipid metabolism-related molecules/substances

Oxysterol-binding protein (OSBP) has been shown to play a critical role in the non-vesicular transport of cholesterol and phosphatidylinositol 4-phosphate (PI4P) between the endoplasmic reticulum (ER) and the Golgi apparatus. OSBP is recruited to viral replication organelles (ROs) through PI4KB-mediated accumulation of PI4P, where it facilitates the delivery of cholesterol to stabilize the structural integrity of ROs and establish a lipid environment essential for viral RNA replication and the assembly of viral replication complexes (79, 80). Itraconazole (ITZ), a clinically approved antifungal agent with emerging anticancer activity, has recently been identified as a novel inhibitor of OSBP. ITZ directly binds to OSBP at ROs, disrupting its lipid transfer function and thereby suppressing viral genome replication (81). In a murine model of RV infection, intranasal administration of itraconazole significantly reduced viral loads and attenuated the release of pro-inflammatory mediators associated with infection, highlighting the therapeutic potential of locally delivered ITZ for the prevention or treatment of RV infections (82). However, ITZ has the potential safety risk of prolonging the QT interval and triggering malignant arrhythmias (83–85). Therefore, a comprehensive and rigorous safety assessment must be conducted, especially for patient groups with underlying diseases and those taking other medications such as methadone. In addition, due to its strong hydrophobicity, conventional aqueous formulations are difficult to maintain a uniform dispersion state, which is a key obstacle in the development of pulmonary inhalation preparations (86). Therefore, future research should prioritize the development of optimized pulmonary-targeted preparations and conduct comprehensive toxicological studies.

25-Hydroxycholesterol (25OHC) and 27-hydroxycholesterol (27OHC) are endogenous cholesterol metabolites that play critical roles in regulating cholesterol homeostasis, inflammatory responses, and tumorigenesis. Several of these regulatory pathways are implicated in the innate immune response to viral infections (87–89). As a ligand of OSBP, 25OHC induces the relocalization of OSBP to the Golgi apparatus within cells and reduces the level of endogenous PI4P in the Golgi apparatus (90). Studies employing cloning or serial passage approaches have shown that neither 25OHC nor 27OHC can be selected for aerobic steroid-resistant variants of HRV, indicating that these hydroxycholesterols are unlikely to promote viral resistance during HRV inhibition (91). Moreover, *in vitro* studies using three-dimensional, fully reconstructed human nasal mucosa and bronchial epithelial models derived from cystic fibrosis patients have demonstrated that 27OHC significantly reduces HRV viral titers and protects against virus-induced ciliary damage (91). It should be noted that localized high concentrations of oxygenated steroids can disrupt cellular cholesterol homeostasis, potentially affecting key physiological processes in non-infected cells, such as the synthesis of pulmonary surfactant, lipid raft structure and signal transduction (92, 93). Although 27OHC provides a highly promising proof of concept for targeted host lipid therapy, particularly for high-risk groups such as patients with cystic fibrosis, its clinical application still requires further validation.

Fatty acid synthase (FAS) inhibitors are a pivotal enzyme in the fatty acid biosynthesis pathway that catalyze the conversion of malonyl-CoA, generated by acetyl-CoA carboxylase 1 (ACC1), into palmitic acid (94). Palmitic acid serves as a fundamental building block for the synthesis of more complex fatty acids, for the formation of plasma membrane structures, and for the post-translational palmitoylation of both host and viral proteins. The FAS inhibitor TVB-3166 has been shown to significantly reduce RSV progeny production both *in vitro* and *in vivo* and demonstrates a broad-spectrum antiviral activity against other respiratory pathogens, including HRV and parainfluenza virus (PIV) (95). However, systemic inhibition of FAS may lead to hypoglycemia, weight loss, and hepatic steatosis. These have been confirmed in zero-fat-fed FASKOL (FAS knockout in liver) mice (96). Therefore, subsequent studies can prioritize developing inhalation administration methods to reduce systemic exposure and rigorously evaluate their impact on host physiological functions before conducting clinical trials.

HMG-CoA reductase inhibitors, commonly known as statins, are widely prescribed and effective agents for the management of dyslipidemia. In addition to their lipid-lowering properties, statins have been found to modulate innate immune responses by inhibiting or activating NLRP3 inflammasomes and Toll-like receptors (TLRs) (97). Furthermore, statins can suppress STAT1 phosphorylation in the JAK/STAT signaling pathway, thereby interrupting downstream signal transduction (98). For instance, simvastatin, a commonly used statin, could exhibit dose-dependent inhibition of the release of α-interferon, chemokine CXCL10, and phosphorylated signal transducer and activator of transcription protein 1 (pSTAT1) induced by RV in peripheral blood monocytes isolated from patients with respiratory allergy or persistent asthma, thereby attenuating the inflammatory response during RV infection (99). Statins are also effective against RSV, but unlike RV, statins disrupt the dynamic balance of the cytoskeleton by inactivating Rho GTPases, thereby directly inhibiting the infectivity of RSV on cells (100). The current positive results all come from *in vitro* cell models, and there is a lack of relevant animal models and large-sample RCTs to confirm the overall clinical benefits of this treatment for RV infections. At the same time, safety issues such as statin-related muscle symptoms also need to be paid attention to (101).

### 3.2 Nuclear proteins

As the primary pathogen responsible for the common cold, RV replicates in the host cytoplasm, which depends on nuclear host proteins to support efficient proliferation. Splicing factor proline/glutamine-rich (SFPQ), also known as PSF, is an abundant and essential nucleoprotein that functions as a member of the DBHS protein family and participates in diverse gene regulatory processes and cellular response pathways (102, 103). Recently, SFPQ has been identified as a key proviral factor for RV replication. During HRV16 infection of HeLa cells, the viral 3CD protease translocates into the nucleus, cleaves SFPQ at glutamine Q257, and generates a C-terminal fragment harboring two RNA recognition motifs (RRMs). This fragment is then exported to the cytoplasm via impaired nuclear pore complexes, where it binds viral RNA to promote viral replication and assembly, thus increasing viral titer and replication efficiency (104).

This process reveals a conserved strategy employed by cytoplasmic RNA viruses to hijack nuclear proteins, highlighting a promising target for broad-spectrum therapies against RV and related enteroviruses (e.g., poliovirus and coxsackievirus). Potential interventions include preserving nuclear pore integrity or disrupting specific host-virus interactions. Although this conserved nuclear hijacking mechanism provides a potential direction, its translational application feasibility is still low. Firstly, its efficacy in primary airway cells and animal models has not been verified. Secondly, targeting SFPQ is a safety hazard, and highly specific interventions (such as blocking 3CD protease cleavage) are needed to avoid cytotoxicity. Moreover, *in vivo* proof of concept studies and clinical trial data are completely lacking. Therefore, until the above shortcomings are addressed, this mechanism only has important biological research value and cannot be used as a feasible therapeutic target.

### 3.3 Cellular helicases

RNA viruses depend on the host’s cellular translation machinery to produce substantial quantities of viral proteins. Most mammalian mRNAs initiate translation through cap-dependent mechanisms. The recognition of the 5’ cap is mediated by eIF4F, which is a complex composed of eIF4E (the actual cap-binding protein), eIF4A (an ATP-dependent RNA helicase), and eIF4G (a large scaffold protein) (105). The cap-binding complex eIF4F recruits the 43S complex to form the 48S translation initiation complex, which then scans along the mRNA until it encounters the start codon, initiating translation (106).

The recent progress in developing compounds targeting the host-dependent DEAD-box helicase eIF4A highlights the considerable potential of exploiting this host factor as a broad-spectrum antiviral strategy (107).

Rocaglates are a class of plant-derived diterpenoid compounds that exert potent antiviral effects by stabilizing mRNA molecules with highly structured 5’ untranslated regions (5’UTRs) on the eIF4A surface through specific stacking interactions. This stabilization impedes the assembly of translation initiation complexes, thereby effectively suppressing viral protein synthesis across a range of RNA viruses (108). Silvestrol is a rocaglate compound isolated from the Asian rosewood plant of the Aglaia genus. In HRV-A1-infected MRC-5 cells, its half-maximal effective concentration (EC_50_) was determined to be 100 nM. However, compared with coronaviruses, HRV-A1 is about 30 to 80 times less sensitive to silvestrol (109). This difference in sensitivity is most likely due to the structural complexity of the 5’UTRs of the different viruses, since coronaviruses have long and complex 5’UTRs (110, 111). A multicenter Phase 1b trial is ongoing to evaluate intravenous and subcutaneous formulations of zotatifin (eFT226), a small-molecule eIF4A inhibitor, in adult patients with mild-to-moderate COVID-19 (112). However, there is still a lack of animal models and clinical studies of eIF4A inhibitors against RV infection at present, and the safe dose and potential toxicity are not clear.

### 3.4 Mitogen-activated protein kinase pathway

A recent study has identified a shared mechanism of action for the CXCL8/MAPK/hnRNP-K signaling axis in host responses to multiple respiratory viruses, including RV, influenza virus, EV-D68, and SARS-CoV-2. Viral infection triggers CXCL8 secretion via specific signaling pathways. CXCL8 acts through CXCR1/CXCR2 receptors to activate the MAPK pathway, causing cytoplasmic translocation of hnRNP-K. Cytoplasmic hnRNP-K binds RV 5’UTRs to facilitate assembly and function of viral RNA replication complexes (113). This indicates that targeting the CXCL8-mediated MAPK signaling pathway holds potential therapeutic value in the treatment of various respiratory viral infections.

MEK1/2 inhibitors represent a class of therapeutic agents that suppress the activity of mitogen-activated protein kinases 1 and 2 (MEK1 and MEK2), which are key enzymes within the RAS/RAF/MEK/ERK signaling cascade. MEK1/2 inhibitors, including selumetinib, trametinib, and CI-1040 (PD 184352), have been approved for use in cancer therapy and are increasingly investigated for their antiviral potential (114). Theoretically, targeting this conserved host pathway offers a high genetic barrier to resistance compared with DAAs, because viruses cannot readily mutate host proteins.

Moreover, the combination of MEKIs with DAAs has shown promise in enhancing antiviral efficacy. While certain MEKIs (e.g., zaponretinib) have entered clinical evaluation for the treatment of COVID-19 and influenza (115), research on the application of MEKIs against RV remains sparse. It is also important to note that the MAPK signaling pathway regulates multiple essential physiological processes, such as cell proliferation, differentiation, and survival (116). Consequently, chronic administration or high-dose suppression of this pathway may lead to dermatological toxicities, including rash and acneiform dermatitis (117), as well as gastrointestinal disturbances such as nausea, vomiting, and diarrhea (118). To mitigate these risks, future investigations could focus on inhaled formulations of MEKIs, which may achieve therapeutic concentrations locally in the respiratory tract while minimizing systemic exposure.

### 3.5 Phosphoinositide kinases

Lipid phosphatidylinositol plays a fundamental role in nearly all pathways regulating cell growth, migration, division, and death, acting as a master regulator of virtually every aspect of cellular life and survival (119). Phosphoinositide kinases (PIKs) catalyze the phosphorylation of phosphatidylinositol to generate phosphoinositides. Mammals express 19 PIK isoforms, which are classified into three major families: phosphoinositide-3-kinase (PI3K), phosphoinositide-4-kinase (PI4K), and phosphoinositide-phosphate kinase (PIPK) (120). Dysregulation of phosphoinositide kinases has been implicated in a wide range of human diseases, including cancer, viral infections, neurodegenerative disorders, developmental abnormalities, and diabetes (121).

Enviroxime-like compounds have been found to inhibit the lipid kinase phosphatidylinositol 4-kinase IIIβ (PI4KIIIβ), thereby suppressing RV replication; however, these compounds exhibit dose-dependent cytotoxicity (122). Accumulating evidence further highlights the critical involvement of the PI3K/Akt signaling pathway in RV infection. For instance, LY294002 has been demonstrated to block Akt phosphorylation and reduce the colocalization of RV39 with Akt (123). RV activates the PI3K-mTOR signaling axis to the upregulation of multiple proinflammatory cytokines, including IL-6 and IL-8. Pharmacological inhibition of this pathway can attenuate the associated inflammatory response with potential therapeutic benefits in conditions of asthma or RV-induced acute exacerbations of chronic obstructive pulmonary disease (124). However, targeting the PI3K-mTOR pathway faces an “immunological paradox”. This pathway is essential for host antiviral immunity, and its non-selective inhibition may impair antiviral defense, leading to elevated viral loads and prolonged viral shedding (125, 126). Since both excessive inflammatory suppression and viral clearance are regulated by the same signaling nodes, achieving a proper therapeutic balance is challenging. Furthermore, systemic administration of mTOR inhibitors such as rapamycin and its derivatives causes metabolic abnormalities, including elevated total cholesterol, LDL cholesterol, and triglycerides, which are particularly prominent in elderly patients with chronic diseases (127). Although PI3K-mTOR inhibitors have potential value for treating inflammatory lung disease in high-risk patients, there is insufficient clinical evidence to support their routine use. Future research should define a precise therapeutic window and explore local delivery strategies to reduce systemic immunosuppression and metabolic side effects.

EV-A71, an enterovirus of the same genus and family as RV, could enhance viral replication by activating the PI3K/Akt signaling pathway, which in turn upregulates key glycolytic proteins such as Glut1. Inhibition of glycolysis using 2-deoxy-D-glucose (2-DG) or oxamate could significantly suppress EV-A71 replication (128). Similarly, 2-DG was found to markedly inhibit RV-B14 replication in both HeLa cells and primary human fibroblasts, and also reduce inflammation and viral load in mouse models of RV airway infection (129). In a recent Phase I study, intranasal administration of 2-DG showed favorable tolerability and safety profiles, with negligible systemic exposure. Meanwhile, the peak drug concentration in the nasal cavity was comparable to the concentration level at which antiviral activity against RV has been demonstrated *in vitro* (130). In conclusion, these findings indicate that the PI3K/Akt pathway facilitates the replication of small RNA viruses, including RV, via glycolytic reprogramming, highlighting its potential as an antiviral target. Future strategies must balance effective viral suppression with the minimization of host metabolic toxicity.

## 4 Immunomodulatory strategies

Even though the host immune response triggered by RV infection is essential for controlling viral replication, it is also the primary driver of clinical symptoms and tissue damage. Consequently, rational immunomodulatory strategies that balance effective antiviral immunity by suppressing excessive inflammatory responses are necessary.

The host response to RV infection involves recognition of viral RNA by endosomal Toll-like receptors (TLRs) 3 and 7/8, and cytoplasmic RNA helicases—retinoic acid-inducible gene I (RIG-I) and melanoma differentiation-associated gene 5 (MDA5) (131). RIG-I recognizes the 5′end of viral RNA in its monomeric form and triggers antiviral type I/III interferons (IFNs) via the mitochondrial antiviral signaling protein(MAVS) (132, 133). Both *in vivo* and *in vitro* studies have shown that in bronchial epithelial cells from asthma patients, RV is predominantly sensed by the RIG-I helicase, resulting in enhanced recruitment of the protein ASC, inflammasome oligomerization, and caspase-1 activation. This RIG-I-mediated inflammasome activation promotes the processing and release of mature IL-1β, thereby exacerbating detrimental inflammatory responses. Notably, inhibition of caspase-1 with YVAD or suppression of inflammasome assembly could lead to a twofold increase in IFN-β expression and a 30% elevation in CXCL10 (an interferon-stimulated gene marker) secretion in human bronchial epithelial cells (HBECs) derived from asthmatic individuals (134). These findings suggest that excessive activation of the RIG-I inflammasome pathway in asthma impairs RIG-I-dependent type I and type III interferon responses and downstream ISG expression, ultimately compromising viral clearance and delaying resolution of airway inflammation.

Given that respiratory infections and associated inflammation within the bronchial epithelium, targeted therapeutic interventions such as inhaled inhibitors of the RIG-I inflammasome (inhaled caspase-1 inhibitors or peptides blocking the RIG-I–ASC interaction) may represent a promising novel strategy for managing acute asthma exacerbations and complications arising from viral infections. However, while restoring the interferon response, excessive inhibition of the inflammasome may increase the risk of secondary infections caused by weakened innate immune function. Moreover, the safety of RIG-I inflammasome inhibitors also needs to be considered. Therefore, although RIG-I regulators have antiviral potential, their development still requires rigorous validation in *in vivo* studies that conform to physiological characteristics.

Olfactomedin-like 3 (OLFML3), also known as hOLF44, is a secreted glycoprotein composed of 406 amino acid residues and is one of five human OLFML proteins (135). Although studies have implicated OLFML3 in tumorigenesis (136, 137), its role in viral immunity remains poorly understood. OLFML3, initially identified via a CRISPR screen as an RV-inducible host factor, functions as a potent negative regulator of type I IFN signaling. In *in vitro* cellular models, loss of OLFML3 was shown to markedly reduce RV replication (138), yet these data are limited to immortalized cell lines and lack primary tissue or *in vivo* validation. Distinct from classical viral entry receptors, OLFML3 does not mediate viral attachment or internalization. On the contrary, RV induces the secretion of OLFML3, thereby upregulating SOCS3 expression, which blocks STAT1/STAT2-dependent type I interferon signaling, enabling the virus to evade host innate immune restriction and enhance viral replication (138). Nonetheless, many translational limitations temper its therapeutic potential. Currently, no selective OLFML3 inhibitors or virus-specific neutralizing antibodies have been developed, and the functional epitopes of OLFML3 remain unclear. Given these unresolved uncertainties, comprehensive profiling of OLFML3’s functional epitopes and downstream signaling networks is essential to overcome translational hurdles and support rational anti-RV drug development targeting this immune antagonist.

## 5 Clinical translation challenges and future directions

To date, only a small proportion of FDA-approved antiviral drugs are based on the modulation of host factors (139). Although HTAs against RV demonstrate considerable promise in preclinical studies, their translation into clinical practice encounters multiple challenges. Overcoming these obstacles necessitates multidisciplinary collaboration and the development of innovative strategies.

### 5.1 Complexity in screening and validating host-targeting factors

Whole-genome screening frequently yields an overwhelming number of candidate targets, with extensively cross-coupled regulatory networks that complicate the discrimination between viral replication-specific essential effects and general cellular housekeeping functions (138). Moreover, the lack of precise broad-subtype validation makes it challenging to select antiviral targets with pan-serotype coverage against all RV strains. Encouragingly, recent advances have demonstrated the successful application of small interfering RNA (siRNA), clustered regularly interspaced short palindromic repeats (CRISPR) technology, and small-molecule chemical inhibitors targeting the cellular “motility complex” to identify host cytokines that support viral replication but are non-essential for cellular homeostasis (140). Furthermore, state-of-the-art deep learning frameworks have shown the clear advantages of the coarse-to-fine feature extraction pipelines. Built on position-aware focal attention mechanisms, such models can effectively filter noise in protein residues and improve predictive sensitivity toward unseen drug–target pairs. This supports faster target mining for novel HTAs (141). Nevertheless, given the substantial discrepancies between *in vitro* cell models and the authentic human respiratory microenvironment, conventional immortalized cell lines fail to fully recapitulate the differentiated state of the airway epithelium, the mucus barrier function, and the local immune milieu. Consequently, targets identified during early-stage screening are highly prone to loss of efficacy when validated in primary airway epithelial cells or organoid models.

### 5.2 Drug delivery, tissue-specific barriers, and biosafety risks

Efficient delivery systems are essential for the success of HTAs. Currently explored delivery strategies include lipid nanoparticles (LNPs), such as those employed in COVID-19 mRNA vaccines (142); viral vectors, particularly adeno-associated virus (AAV) vectors (143); and biodegradable, low-toxicity carriers such as peptides, oligopeptides, and peptide-polymer hybrids (144). Inspired by recent success in macrophage-targeted nanomedicine for chronic inflammatory diseases (145), HTAs against RV infection could adopt a similar “immune reprogramming” strategy. The use of functionalized nanocarriers to deliver signaling pathway inhibitors to respiratory epithelial cells or alveolar macrophages holds promise for abrogating virus-induced cytokine storms while preserving the host’s basal immune defense functions. Furthermore, drawing upon tumor microenvironment-responsive release mechanisms, the development of smart nanomedicines sensitive to the respiratory inflammatory microenvironment represents a key direction for enhancing local drug concentrations and minimizing systemic toxicity.

Key challenges in the clinical translation of these delivery systems encompass achieving favorable biodistribution, high transfection efficiency, and excellent biosafety profiles (146, 147). For example, complex physiological barriers (such as respiratory mucosal barriers) can significantly hinder the accumulation of therapeutic agents in target organs, while unintended deposition in non-target tissues may lead to off-target effects (148). This has also been verified in the latest research on the treatment of SARS-CoV-2 infection: Many compounds that are highly effective at the biochemical level fail to penetrate the host cell membrane effectively, are expelled by efflux pumps (such as P-gp, BCRP), or are metabolically inactivated within the cells, resulting in the intracellular unbound drug concentration being much lower than the effective inhibitory concentration (149). Recently, it has been reported that introducing methyl, cyclization, fluorination, and stereoregular positional-blocking modifications to seal the hydroxyl metabolic soft sites significantly improves the stability of small molecules while retaining dual-target inhibitory activity (150). This provides an optimized solution for the rational design of broad-spectrum oral/inhalation candidate molecules for anti-RV.

Since HTAs act on the body’s inherent functional proteins rather than virus-specific proteins, interference with these physiological functions is the core safety concern. Moreover, the prolonged expression mediated by AAV vectors raises concerns about potential genomic integration (151). Traditional short-term *in vitro* toxicity assessment fails to reflect the chronic toxicity resulting from long-term repeated use or seasonal preventive use. It lacks long-term human safety data, which severely hinders the progress to later clinical trials. Addressing these challenges necessitates rigorous *in vitro* and *in vivo* toxicity evaluations, along with comprehensive process optimization to ensure safe and effective delivery.

### 5.3 The narrowness of the treatment window period

The early stage of RV infection (within 24 to 48 hours after infection) is the peak period for viral replication. In the later stage, it is mainly characterized by host inflammatory damage (152). In addition, there is also the problem of diagnostic lag. By the time patients exhibit obvious symptoms, the optimal intervention window has already passed (153). At the same time, RV infection is mostly a mild, self-limiting disease. Healthy individuals may not require antiviral treatment. They are more likely to be applicable to high-risk subgroups such as asthma, COPD, and infants, and it is difficult to establish a unified dosing regimen and dosage standard, thereby complicating the design of clinical trial endpoints and the evaluation of therapeutic efficacy (154).

### 5.4 Issues of individual differences and population heterogeneity

People with asthma and allergic constitution may have variations in susceptible genes such as CDHR3 (63, 64), and there may be individual differences in drug reactions, efficacy, and side effects. There are significant differences in drug-metabolizing enzyme activity, mucosal barrier function, immune response levels, and tolerated doses among infants, the elderly, immunosuppressed individuals, and young adults (155, 156).

The data from the general population cannot be directly applied to these groups. Moreover, the dynamic changes in seasonal RV subtypes (157), as well as differences in nasal and pulmonary microbiota (158), may alter the host microenvironment and the drug-action environment, resulting in large heterogeneity and poor reproducibility of clinical trial efficacy results, and increasing the difficulty of clinical trial design.

**Figure 2:**
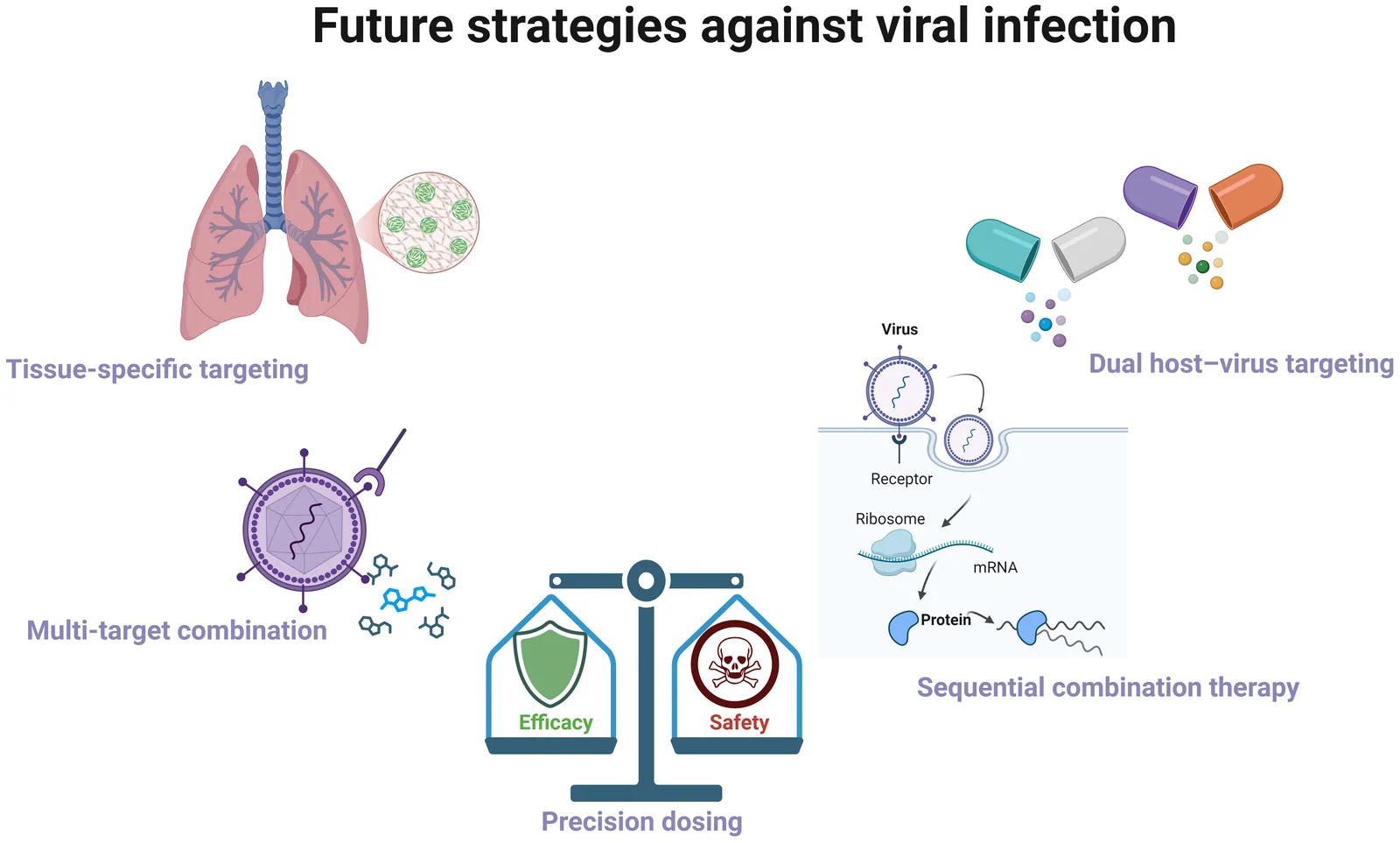
Future strategies against viral infection. The schematic diagram was created using BioRender.com.

In summary, to enhance the therapeutic efficacy of HTAs, future strategies may include (**Figure 2**):

1. Tissue-specific targeting: Employing nanocarriers or adeno-associated viruses (AAVs) for respiratory tract-specific drug delivery to minimize systemic exposure.
2. Multi-target combination: Concurrently modulating multiple host factors to restrict the virus’s capacity to exploit alternative replication pathways.
3. Precision dosing: Defining optimal therapeutic concentration windows that effectively suppress viral replication while preserving essential physiological functions.
4. Sequential combination therapy: Administering agents that target distinct host factors at specific stages of the viral life cycle.
5. Dual host–virus targeting: Integrating host-targeted therapeutics with direct-acting antivirals to maximize antiviral efficacy and establish higher genetic barriers to resistance.

## 6 Conclusion

**Figure 3:**
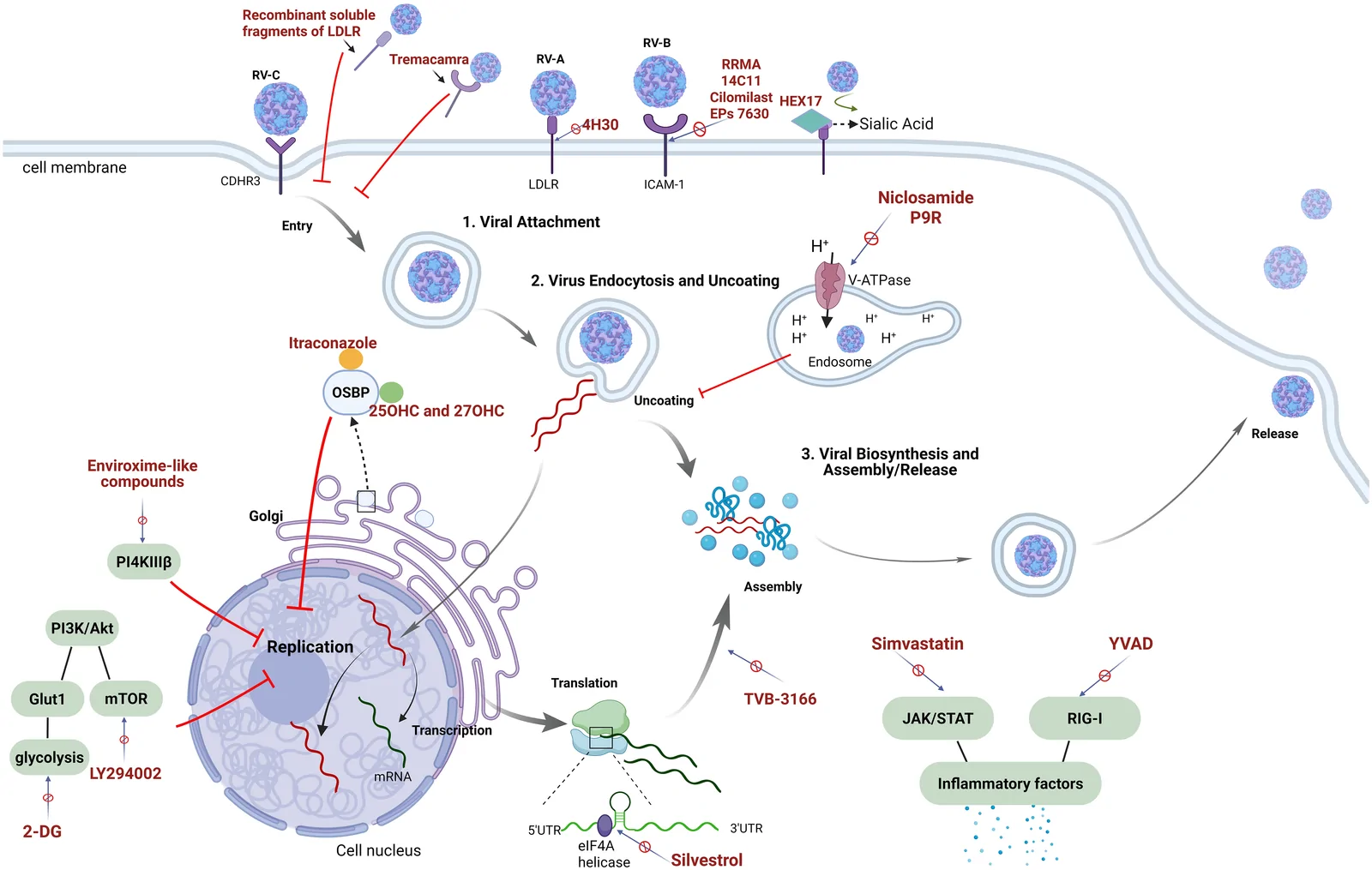
Antiviral strategies targeting key steps in the replication cycle of rhinovirus. RRMA and 14C11 block viral entry by targeting cellular ICAM-1. Cilomilast and EPs 7630 downregulate ICAM-1 expression to hinder viral attachment. Tremacamra and recombinant soluble LDLR fragments function as decoy receptors to intercept viruses and prevent entry. 4H30 targets LDLR, and HEX17 interacts with both ICAM-1 and LDLR to suppress viral entry. Niclosamide and P9R inhibit endosomal acidification, thereby blocking viral uncoating and genomic RNA release. Itraconazole, 25OHC, and 27OHC interact with OSBP to suppress viral replication. TVB-3166 inhibits viral protein synthesis. Simvastatin alleviates virus-triggered inflammatory responses via suppressing STAT1 phosphorylation in the JAK/STAT pathway. Silvestrol impedes the assembly of translation initiation complexes to exert antiviral effects. Enviroxime-like compounds, LY294002 and 2-DG, restrain viral replication by inhibiting PI4KIIIβ, blocking Akt phosphorylation, and suppressing glycolysis, respectively. YVAD relieves virus-induced inflammation through inhibiting excessive activation of the RIG-I inflammasome pathway. These antiviral drugs can specifically interfere with multiple essential steps in the rhinovirus replication process, including rhinovirus adsorption, invasion, uncoating, biosynthesis, assembly, and release, to exert antiviral effects. The schematic diagram was created using BioRender.com.

RV infection has imposed a heavy burden on global public health. HTAs that target highly conserved host cytokines involved in the viral life cycle and immune regulation offer potential avenues for antiviral prevention and treatment (as summarized in **Figure 3**). However, issues such as host toxicity, off-target effects, and individual differences have hindered the clinical translation and application of this therapy. Therefore, subsequent research should prioritize clarifying the complex virus-host interaction network and optimizing local delivery systems to broaden the therapeutic safety window. While HTAs hold theoretical promise, particularly for mitigating exacerbations in high-risk populations, their realization as viable clinical interventions depend on rigorously addressing these safety and delivery challenges.
